# Utilization of Cardiovascular Magnetic Resonance Imaging for Resumption of Athletic Activities Following COVID-19 Infection: An Expert Consensus Document on Behalf of the American Heart Association Council on Cardiovascular Radiology and Intervention Leadership and Endorsed by the Society for Cardiovascular Magnetic Resonance

**DOI:** 10.1161/CIRCIMAGING.122.014106

**Published:** 2022-12-21

**Authors:** Frederick L. Ruberg, Aaron L. Baggish, Allison G. Hays, Michael Jerosch-Herold, Jiwon Kim, Karen G. Ordovas, Gautham Reddy, Chetan Shenoy, Jonathan W. Weinsaft, Pamela K. Woodard

**Affiliations:** 1 Section of Cardiovascular Medicine, Department of Medicine, Boston University School of Medicine/Boston Medical Center, Boston, MA (F.L.R.).; 2 Cardiac Performance Program, Harvard Medical School/Massachusetts General Hospital, Boston, MA (A.L.B.).; 3 Division of Cardiology, Department of Medicine, Johns Hopkins University, Baltimore, MD (A.G.H.).; 4 Cardiovascular Imaging Section, Harvard Medical School/Brigham and Women’s Hospital, Boston, MA (M.J.-H.).; 5 Division of Cardiology, Department of Medicine, Weill Cornell Medicine/New York Presbyterian Hospital, New York, NY (J.K., J.W.W.).; 6 Department of Radiology, University of Washington School of Medicine, Seattle, WA (K.G.O., G.R.).; 7 Cardiovascular Division, Department of Medicine, University of Minnesota Medical School, Minneapolis, MN (C.S.).; 8 Mallinckrodt Institute of Radiology, Washington University School of Medicine, Saint Louis, MO (P.K.W.).

**Keywords:** athlete, COVID-19, magnetic resonance imaging, myocardial infarction, myocarditis

## Abstract

The global pandemic of COVID-19 caused by infection with SARS-CoV-2 is now entering its fourth year with little evidence of abatement. As of December 2022, the World Health Organization Coronavirus (COVID-19) Dashboard reported 643 million cumulative confirmed cases of COVID-19 worldwide and 98 million in the United States alone as the country with the highest number of cases. Although pneumonia with lung injury has been the manifestation of COVID-19 principally responsible for morbidity and mortality, myocardial inflammation and systolic dysfunction though uncommon are well-recognized features that also associate with adverse prognosis. Given the broad swath of the population infected with COVID-19, the large number of affected professional, collegiate, and amateur athletes raises concern regarding the safe resumption of athletic activity (return to play) following resolution of infection. A variety of different testing combinations that leverage ECG, echocardiography, circulating cardiac biomarkers, and cardiovascular magnetic resonance imaging have been proposed and implemented to mitigate risk. Cardiovascular magnetic resonance in particular affords high sensitivity for myocarditis but has been employed and interpreted nonuniformly in the context of COVID-19 thereby raising uncertainty as to the generalizability and clinical relevance of findings with respect to return to play. This consensus document synthesizes available evidence to contextualize the appropriate utilization of cardiovascular magnetic resonance in the return to play assessment of athletes with prior COVID-19 infection to facilitate informed, evidence-based decisions, while identifying knowledge gaps that merit further investigation.

COVID-19 infection can result in a varying severity of manifestations affecting multiple organ systems. Although respiratory illness is the most common clinical manifestation of COVID-19, cardiovascular involvement can also occur. Cardiovascular manifestations associated with COVID-19 include myocardial infarction,^1^ myocarditis,^2^ arrhythmia,^3^ and stress cardiomyopathy.^4^ Cardiac biomarker (troponin) elevation is a commonly reported abnormality in COVID-19, occurring in 20% to 36% of patient hospitalized with COVID-19, and is associated with greater disease severity including need for mechanical ventilation and increased risk of death.^5,6^ The underlying pathophysiologic mechanism of troponin elevation is incompletely understood and is likely multifactorial in etiology resulting from systemic illness and upregulation of systemic inflammatory and prothrombotic pathways.^7,8^ Although myocarditis may be suspected in patients with elevated cardiac biomarkers and there is an association between COVID-19 infection and myocarditis, it is important to note that direct viral infection of the myocardium caused by COVID-19 has been uncommonly confirmed by histological analyses.^9^ For example, in an autopsy study of 39 COVID-19 infected patients in Germany, 62% had evidence of the viral genome within the heart, though findings did not meet histopathologic criteria (ie, inflammatory infiltrate) for myocarditis.^10^ That said, a more recent report did convincingly show evidence of cardiomyocyte COVID-19 infection with resultant cardiac injury and increased macrophage abundance.^11^ In this context, although prior studies have shown troponin elevation to correlate with severity of illness and extent of COVID-19 viremia,^7,12,13^ it is unknown whether troponin release simply mirrors disease severity or has mechanistic implications for worsened prognosis.

Regarding severity of illness, COVID-19 infection can result in a wide spectrum of disease manifestations ranging from no symptoms to critical illness and can be grouped into the following categories^14^: (1) asymptomatic or presymptomatic (no signs or symptoms of infection despite positive SARS-CoV-2 virological test), (2) mild illness (upper respiratory infection and other mild symptoms without shortness of breath or abnormal chest imaging), (3) moderate illness (lower respiratory disease on clinical or imaging assessment and SpO_2_ ≥94%), (4) severe illness (SpO_2_<94%, PaO_2_/FiO_2_ <300 mm Hg, respiratory rate >30 breaths/min, or lung infiltrates >50%), and (5) critical illness (respiratory failure, septic shock or multiple organ dysfunction). Whereas some COVID-19 survivors recover quickly, others have a more prolonged course of illness due to persistent symptoms (long COVID syndromes which are now collectively referred to as postacute sequelae of SARS-CoV-2 infection [PASC]).^15^ For example, among 143 patients with resolved COVID-19 (2 negative polymerase chain reaction tests) who required hospitalization, 87% had at least one ongoing cardiopulmonary symptom—including fatigue (53%), dyspnea (43%), and chest pain (22%), and nearly half (44%) had worsened quality of life at 60 days after acute infection.^16^ Although wide variability in time to symptom resolution has been reported, recovery time appears to be associated with preexisting risk factors as well as severity of acute COVID-19 illness.^16-18^

## Imaging of Cardiac Involvement

Cardiovascular imaging plays an important role in the evaluation of COVID-19 patients with suspected cardiac involvement. Transthoracic echocardiogram (TTE) evidenced left and right ventricular (LV) and (RV) dysfunction has been commonly reported in acute COVID-19 infection requiring hospitalization, occurring in up to 41% and 15% of affected patients, respectively.^19^ Such TTE evidence of ventricular contractile dysfunction during acute infection has been shown to provide incremental prognostic utility to clinical and biomarker indices. In convalescent patients with COVID-19, patients with LV and RV dysfunction on initial TTE have shown improvement following recovery from acute illness.^20,21^ For example, on prospective TTE longitudinal follow-up of 79 hospitalized patients with COVID-19 pneumonia, prevalence of RV and LV abnormalities decreased from 51% to 19% and 13% to 9% following recovery, respectively.^21^ Similarly, in the World Alliance Societies of Echocardiography-COVID study, patients with impaired LV and RV longitudinal strain at baseline had significant improvement on follow-up (LV: −14.5%±2.9% versus −16.7%±5.2%, *P*<0.001 and RV: −15.2%±3.4% versus −17.4%±4.9%, *P*=0.004, respectively),^20^ supporting the general notion that the acute functional decline associated with infection is reversible in some patients in whom dynamic changes may be attributable to hemodynamic or other transient consequences of acute systemic illness. Furthermore, there is likely a heterogeneity of pathologies responsible for contractile dysfunction, some of which are irreversible (ie, myocyte necrosis in context of hypoperfusion) and others that are transient (ie, stunning/contractile depression in context of acute systemic illness).

**Figure 1.**
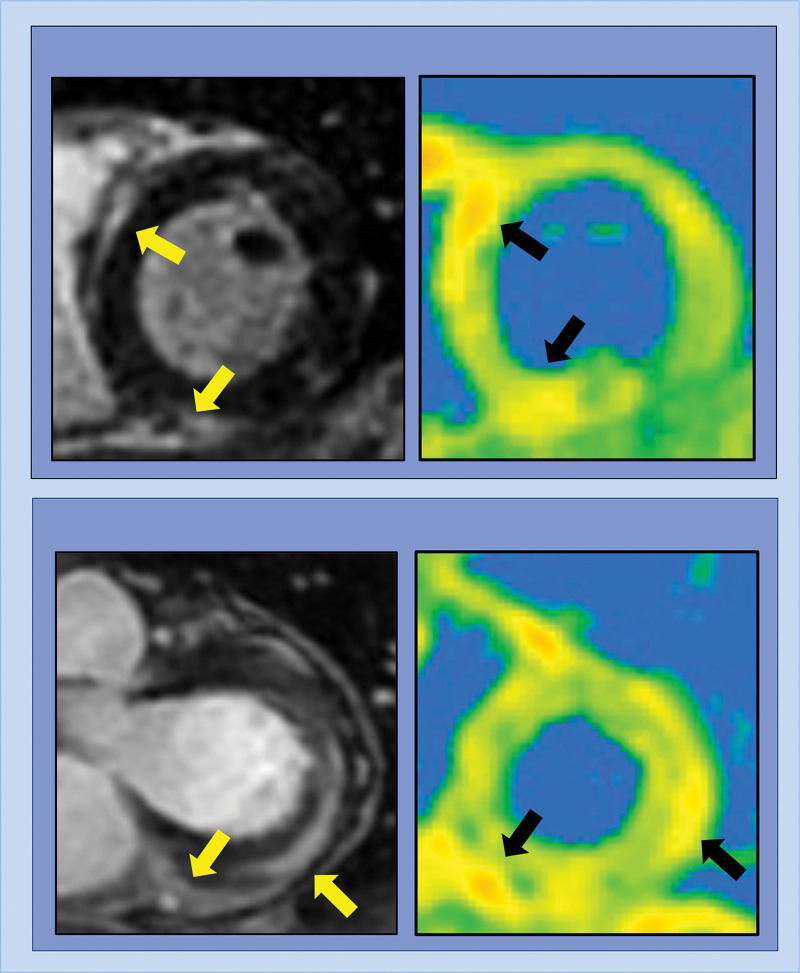
**Representative cardiovascular magnetic resonance (CMR) examples of altered myocardial substrate in patients following acute COVID-19 infection.** Note focal fibrosis (yellow arrows) accompanied by increased T2 (black arrows) on parametric mapping consistent with myocardial edema.

Cardiovascular magnetic resonance (CMR) is uniquely capable of characterizing myocardial tissue properties in vivo, enabling assessment of pattern and functional sequelae of cardiac injury, including evaluation for myocardial edema present in myocarditis (Figure 1). Although CMR exams during acute COVID-19 illness have been less frequently performed owing to associated critical illness and concerns regarding patient monitoring and transmission during prolonged imaging, an initial case report described CMR abnormalities including LV dysfunction, abnormal T2 times consistent with interstitial edema, pericardial effusion, and a nonischemic pattern of diffuse late gadolinium enhancement (LGE).^2^ More recently, CMR abnormalities including impaired LV function via strain and high prevalence of myocardial edema on T2 weighted imaging (56%) have been described in 25 patients with acute COVID-19 infection who underwent imaging within 10 days of initial COVID-19 symptoms.^22^ Supporting findings in prior longitudinal TTE studies that demonstrate functional recovery,^20,21^ in this cohort, there was low prevalence of irreversible myocardial necrosis with only one patient demonstrating LGE (4%).

## Role of CMR in the Evaluation of Suspected Acute Myocarditis

Clinical presentations of acute inflammatory cardiomyopathy or myocarditis include acute coronary syndrome-like presentation, new-onset or worsening chronic heart failure, life-threatening arrhythmia, and cardiogenic shock.^23^ A diagnosis of myocarditis is made using ≥1 diagnostic tests including ECG, blood markers of myocardial injury (troponin-T or -I), endomyocardial biopsy, and cardiac imaging. Common cardiac imaging tests used to diagnose myocarditis include TTE (which can identify functional and anatomic sequelae) and CMR (which can concomitantly identify alterations in myocardial function, anatomy, and tissue properties). CMR is a key test in the contemporary assessment of patients with suspected myocarditis and is often used to establish the diagnosis owing to its unparalleled capacity to characterize myocardial tissue.^23-25^

**Figure 2.**
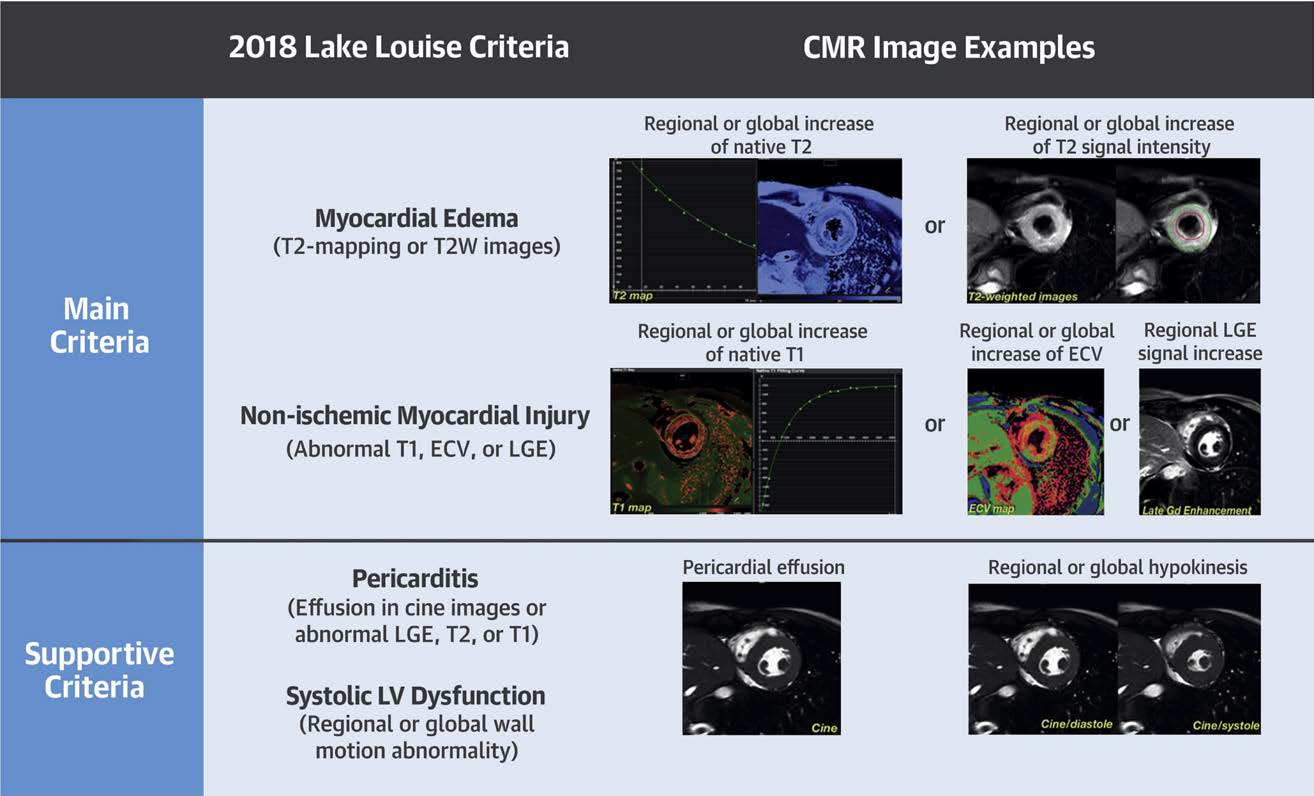
**Overview of the updated Lake Louise Criteria.** Reproduced from Ferreira et al^27^ with permission. Copyright ©2018, Elsevier. CMR indicates cardiac magnetic resonance; ECV, extracellular volume fraction; and LGE, late gadolinium enhancement.

In 2009, an International Consensus Group on CMR Diagnosis of Myocarditis comprised of 22 experts published recommendations (dubbed the Lake Louise Criteria or LLC for where they met in Alberta, Canada) on the indications for CMR, the protocol, and analyses for the diagnosis of myocarditis.^26^ The criteria were revised in 2018 to incorporate evidence that parametric mapping techniques (including native T1 and T2 mapping) could be used to identify myocarditis among patients with sufficient pretest probability. The update was prompted by the development of contemporary CMR mapping techniques, allowing efficient measurement of myocardial T1 and T2 relaxation times and several studies describing high sensitivity, specificity, and diagnostic accuracy of mapping techniques in the CMR assessment of suspected myocarditis.^27^ The use of 3 integrated approaches involves LGE for highlighting focal myocardial injury, T1 mapping to identify diffuse myocardial fibrosis, and T2 mapping to reveal (diffuse) myocardial edema. The JACC Scientific Expert Panel comprised of 11 experts published updated criteria^27^ (Figure 2) recommending that CMR provides strong evidence for acute myocarditis if criteria in each of 2 categories were met:

1. Abnormal T1-based marker for myocardial injury on T1 mapping—abnormal native (noncontrast) T1 or extracellular volume fraction (ECV)—or LGE imaging (in a nonischemic pattern),
2. Abnormal T2-based marker for myocardial edema on T2 mapping or T2-weighted imaging.

## Overview of T1 and T2 Mapping in Acute Myocarditis

T1 and T2 relaxation times are determined by the tissue composition, interstitial, and intracellular milieus, and external factors, such as the magnetic field strength and the methods of measurement, including the hardware and the software platforms. Since T1 and T2 mapping techniques vary by the magnetic field, hardware, and pulse sequences used, the normal ranges of myocardial T1 and T2 values are derived from healthy individuals imaged locally on the same CMR scanner or equivalent systems. Abnormal T1 and T2 relaxation times (ie, outside the normal range) help with the detection and diagnosis of myocardial pathology. Using T1 and T2 mapping techniques, global or regional myocardial T1 or T2 relaxation times can be obtained with pixel-level resolution. Myocardial ECV can be measured using precontrast and postcontrast T1 mapping and incorporating the hematocrit value. Recommendations and challenges in the clinical application of T1 and T2 mapping are covered in detail in a consensus statement by the Society for Cardiovascular Magnetic Resonance endorsed by the European Association for Cardiovascular Imaging.^28^

Abnormal native T1 and ECV suggest an expansion of the extracellular or interstitial space, which could occur globally or regionally due to various pathologies including acute myocardial inflammation and edema, vasodilation, hyperemia, and capillary leak, myocardial necrosis, and myocardial fibrosis. Abnormal native T1 may also reflect intracellular edema.^28^ LGE also depicts extracellular pathology, and unlike native T1 or ECV, it generally reflects irreversible myocardial damage. This could be necrosis with accompanying inflammatory changes such as edema in the acute setting, or fibrosis in the chronic setting. Abnormal T2 is principally a marker for increased water content, either intracellularly or extracellularly, related to inflammation and edema.

## CMR to Diagnose COVID-19 Myocardial Involvement in Athletes

CMR has been widely used to detect COVID-19 myocardial involvement in athletes with either COVID-19 or asymptomatic SARS-CoV-2 infection with great variation in application. The use of CMR has been routine in all athletes at some institutions, whereas others have employed a strategy of limited use of CMR to investigate abnormalities on other tests, such as troponins, TTE, and ECG (termed triad testing). Using the updated LLC, a proportion of athletes have been diagnosed with COVID-19 myocardial involvement based on abnormalities on T1 and T2 mapping, often without LGE. It is important to emphasize that established CMR measures of cine volume and function or myocardial damage as identified by LGE are better validated than the more novel mapping techniques. Thus, the application of CMR and the updated LLC to diagnose COVID-19 myocardial involvement in athletes with either COVID-19 but no symptoms of myocarditis or asymptomatic SARS-CoV-2 infection, and its use in return to play (RTP) decision-making requires nuance because of certain caveats enumerated below.

First, the limited specificity of the T1- and T2-mapping-based criteria in the updated LLC combined with the low prevalence of COVID-19 myocardial involvement in general—and particularly in young, previously healthy athletes—leads to a low positive predictive value for the criteria in this patient group. Second, most patients in the validation studies of myocarditis diagnosed on CMR have LGE, which has been validated more extensively than mapping abnormalities in histologically proven viral myocarditis.^29,30^ Third, although several prognostic studies have established an adverse prognostic significance for LGE in patients with myocarditis,^31-33^ including a study with >10 years of follow-up,^33^ there are no prognostic data for abnormalities on T1 or T2 mapping in the absence of LGE. For ECV, there is at least one study describing its prognostic value in patients with suspected myocarditis, but ECV only maintained an independent association with outcomes independent of LGE at a relatively high ECV threshold (ECV>0.35).^34^ Fourth, the literature validating the use of CMR for myocarditis using pathology and outcome data involves symptomatic patients with inflammation largely limited to the heart. Indeed, there are several articles on abnormalities describing T1 or T2 mapping independent of, or in the absence of, LGE in asymptomatic patients comprising a wide gamut of systemic inflammatory conditions including autoimmune diseases, such as systemic lupus erythematosus^35-37^ or systemic sclerosis,^37-39^ sarcoidosis,^40-42^ or infectious diseases, such as human immunodeficiency virus (HIV).^43,44^ Simultaneously, there are a dearth of data regarding T1 or T2 mapping abnormalities in the absence of LGE from the perspectives of pathology validation, natural evolution over time into cardiac damage in the form of necrosis or fibrosis, or prognostic implications. Finally, although rare, COVID-19 vaccine–related myocarditis shares similar reported CMR features with COVID-19 myocardial involvement in athletes, and occurs almost exclusively in adolescents and young adults,^45-47^ the same demographic as many athletes. Given the reported findings of T1 or T2 mapping abnormalities, without irreversible cardiac damage (LGE) in systemic inflammatory conditions, it is plausible that COVID-19, an infection featuring significant systemic inflammation, could be accompanied by myocardial inflammation without direct cardiac involvement by SARS-CoV-2 or irreversible damage, calling to question the prognostic significance of these findings.

## Summary of Published Studies Describing CMR in the Recovered Athlete

Early during the COVID-19 pandemic, several studies reported a high prevalence of cardiac involvement detected on CMR after recovery from COVID-19, even among patients who were initially asymptomatic or minimally symptomatic during the acute infection.^48-50^ However, there was high variability among studies for presence of cardiac involvement (ranging from 26% to 78% prevalence) and of methods used to quantify and report myocardial tissue characterization. The plethora of early data showing a high prevalence of myocardial abnormalities postinfection led to heightened concern regarding the safety of athletes preparing to RTP after COVID-19 infection. These early observations led to the initiation of clinical CMR studies specifically focused on athletes postinfection to evaluate for myocardial injury.

As the pandemic continued, additional small, single-center observational studies of collegiate and professional athletes undergoing CMR assessment for RTP eligibility post–COVID-19 infection reported variable prevalence of cardiac involvement by CMR (ranging from 0% to 100%, Table 1).^48,51-62^ A more detailed review of the literature can be found in the American College of Cardiology Solution Set Oversight Committee’s Expert Consensus Decision Pathway on Cardiovascular Sequelae of COVID-19 in Adults: Myocarditis and Other Myocardial Involvement, Post-Acute Sequelae of SARS-CoV-2 Infection, and RTP.^63^ Again, in many of these studies, nonstandardized methods were used for CMR-determined cardiac involvement and cardiac abnormalities did not meet LLC for myocarditis. Moreover, in the majority of these published studies, control groups of uninfected athletes were not included as a comparator. Thus, these studies provide little evidence on whether similar findings might be seen in myocardial remodeling in highly conditioned athletes^64^ who had not previously had COVID-19 infection. Moreover, without meeting LLC, many studies reported findings that were not specific to myocarditis. A large study in professional athletes used a tiered testing approach consisting of the commonly employed triad testing of cardiac troponin, 12-lead ECG, and TTE, with CMR performed only when clinically indicated or when suggested by abnormal initial testing. Using this strategy, cardiac involvement was found to be only 0.6%.^65^ Finally, in a study of 147 COVID-19 positive athletes which did have athletic (N=59) and healthy athletic controls (N=56), CMR showed no differences in volumetric, functional, or tissue characteristics between athletes with prior COVID-19 infection and matched healthy athletes. Although 4.7% (n=7) of COVID-19–positive athletes had findings consistent with myocarditis, none were asymptomatic.^61^

**Table 1.**
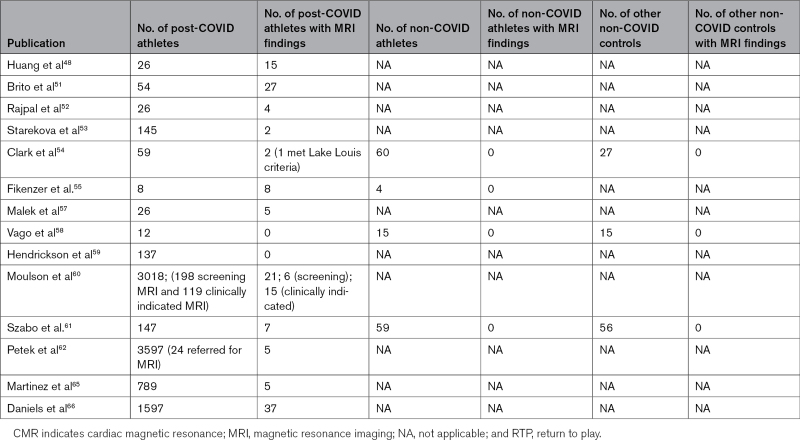
Summary of Published Studies Utilizing CMR in the RTP Assessment of COVID-19 Infected Athletes

More recently, several large cohort studies in athletes used the updated 2018 LLC^27^ to determine cardiac involvement by CMR. Although the LLC was originally developed for the diagnosis of myocarditis in symptomatic patients, these criteria were adapted to ascertain whether athletes had definite, probable, or possible myocarditis post–COVID-19, some of whom were asymptomatic. Important modifications to the LLC criteria included considering supplemental information, such as reduced LV ejection fraction and pericardial involvement more strongly.^60,65^ Also, ensuring that T1 and T2 abnormalities colocalized in the same myocardial region was important in the reduction of variability and improvement of specificity. In a large cohort study of 1,597 athletes (Big Ten COVID-19 Cardiac Involvement registry), Daniels et al^66^ reported a prevalence of 2.3% of clinical and subclinical myocarditis using the modified LLC definitions (Table 1). The most common CMR abnormalities detected were elevated T2 indicative of edema and nonischemic patterns of LGE. However, the authors acknowledged limitations including the lack of standardized timing from COVID-19 infection to cardiac testing (discussed in detail below) and institutional differences in CMR interpretation.

Another large registry study, the ORCCA (Outcomes Registry for Cardiac Conditions in Athletes),^60^ investigated cardiac involvement post–COVID-19 among competitive athletes using the same modified LLC approach. In this study, 198 athletes underwent primary CMR screening whereas another 119 underwent CMR only if clinically indicated per triad noninvasive screening or clinical judgment. The authors reported a low prevalence of cardiac involvement (ranging from 0.5% to 3.0%) and no adverse cardiac events in the short term in over 3000 infected athletes after resumption of normal athletic activities with definite, probable, or possible cardiac involvement. Furthermore, they noted an over 4-fold increase in diagnostic yield when CMR was performed when indicated by triad testing as opposed to widespread screening. Based on these results, the authors concluded that CMR was most useful in athletes with a high pretest probability of cardiac involvement defined by abnormalities on triad testing or the presence of cardiopulmonary symptoms. Although the registry studies overall indicate a low prevalence of COVID-19–related cardiac involvement in athletes, the limitations of the studies include variability of CMR interpretation with no centralized imaging core lab, use of LLC in asymptomatic individuals with unclear clinical implications, and lack of a control group.^60,65,66^

## Application of Triad Testing in the RTP Assessment

Clinical data characterizing hospitalized patients with severe COVID-19 that suggested a high prevalence of cardiac injury as defined by cardiac troponin elevation stimulated concern about athlete safety following COVID-19 infection thereby prompting the development of post-infectious RTP screening protocols.^67,68^ Initial expert consensus recommendations suggested cardiac triad testing (ie, ECG, cardiac troponin, and TTE) for competitive athletes with symptoms following COVID-19 infection.^69^ This conservative approach was presented during the initial global sports hiatus when clinical experience with infected but otherwise healthy athletes was minimal. As summarized above, widespread implementation of triad testing during the subsequent return of organized athletics provided an abundance of data leading to several important scientific advances. Multicenter registry data at both the professional and collegiate levels, including one collegiate registry examining the role of mandatory CMR imaging, demonstrated a lower than anticipated prevalence of clinically relevant post-infectious cardiac involvement (≈0.5%).^60^ In addition, these databases established links between the severity of acute infection, the presence of symptoms during return to exercise, and the likelihood of acute cardiac inflammation. Ongoing research continues to test the prognostic utility of CMR in COVID-19 survivors, including optimal screening algorithms in athletic and nonathletic cohorts, patient profiles that predict increased diagnostic yield for CMR-based testing, and specific findings on CMR (including functional and tissue substrate alterations) most associated with residual clinical symptoms, impaired quality of life, and long-term clinical risk.

These observations have led to refinement of RTP screening protocols with an emphasis on limiting testing to athletes at highest risk for cardiac complications following infection. Although ongoing research is focused on refining risk profiles and screening algorithms, current expert consensus recommendations suggest cardiac testing only among athletes infected with COVID-19 who require hospitalization or those who develop cardiac symptoms (chest pain, syncope, palpitations, dyspnea) during or after the acute phase of infection.^63^ These recommendations are consistent with the clinical approach in any other viral syndrome during which cardiopulmonary symptoms develop raising concern for myocarditis. Symptoms suggestive of acute cardiac inflammation include chest pain at rest or with exertion, subjective or objective tachyarrhythmia, or a heart failure syndrome. Athletes with ≥1 of these symptoms are at moderate or high pretest probability of having acute cardiac inflammation and should undergo comprehensive evaluation before returning to training and competition. This evaluation should include cardiac triad testing with an emphasis on detecting findings related to acute cardiac inflammation (Table 2). The specificity of 12-lead ECG to identify injury patterns^70^ or TTE^71^ to identify altered cardiac structure and function in the context of acute cardiac inflammation will be maximized when compared to preinfection baseline values when available.

**Table 2.**
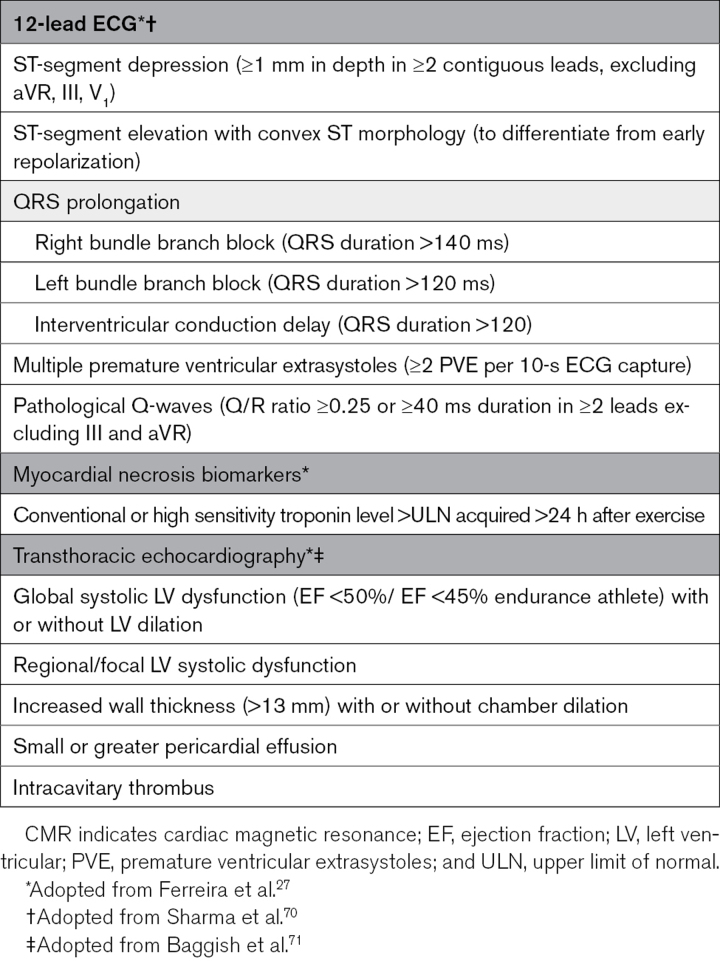
Findings on Cardiac Triad Testing Following COVID-19 Infection That Should Prompt CMR

## Lack of Standardization in CMR Techniques Resulting in Variability in Findings

One key cause of variability in prevalence of cardiac involvement in both athletes and nonathletes post–COVID-19 stems from lack of standardized interpretation of CMR abnormalities. Several different strategies of image analysis have been implemented across COVID-19 CMR studies including reporting (1) any T1, T2, and LGE abnormality detected even in isolation (qualitative visual assessment and mapping), (2) reporting abnormalities according to LLC, and (3) modifying LLC to require colocalization of T1 and T2 abnormalities and including supportive findings, such as pericarditis or reduced LV ejection fraction. Given the heterogeneity of methods of image acquisition, type and field strength of scanners, and variability in interpreting and reporting tissue characterization abnormalities, it is not surprising that high variability of cardiac involvement exists between studies. Furthermore, this highlights the need to better standardize CMR metrics to quantify cardiac involvement in myocarditis, particularly as they pertain to T1 and T2 measures which can be variable. Finally, even in large cohort studies, the number of true myocarditis cases are few. In this context, it is likely that a uniform strategy of CMR screening of all athletes who have recovered from COVID-19 with no clinical findings or symptomatology would result in the identification of a substantial number of cases with abnormal CMR findings, for whom clinical, therapeutic, and long-term prognostic relevance is uncertain. Given the fact that substantial equipoise exists regarding this issue, evidenced-based data are lacking at present to support such a uniform screening strategy. Moreover, to date, there are no outcomes studies in COVID-19–positive athletes with abnormal CMR findings. Thus, based on currently available evidence, these positive CMR findings in asymptomatic athletes (without ancillary testing indicative of contractile dysfunction, electrical, or biomarker alterations) are most likely unhelpful in guiding physicians and coaches alike in determining RTP.

## Technical Considerations Regarding Heterogeneity in LGE and Mapping Techniques

Among imaging modalities applied to studying pathological changes in the heart after COVID-19 infection, CMR has played a prominent role because the relaxographic (ie, T1 and T2) properties of myocardial tissue are relatively sensitive to changes at the cellular and molecular level. Whereas the overarching approach used with CMR for tissue characterization follows a relatively standardized schema to assess patterns of altered myocardial tissue substrate, it is worth noting that each of its components may vary in terms of pulse sequence, parameter settings, and post-processing thereby potentially introducing substantial heterogeneity with respect to prevalence and extent and magnitude of such abnormalities.

Regarding LGE, it is well-established that spatial resolution varies in relation to acquisition scheme, and that improved spatial resolution provides improved scar/fibrosis detection. Among 20 COVID-19 survivors, Bustin et al^72^ reported that focal fibrosis was evident on high-resolution LGE-CMR (isotropic voxel size 0.6cm^3^) in 67% of patients (n=12), among whom conventional LGE-CMR (voxel size 1.5×1.5×4.0 mm) was interpreted as negative or inconclusive in 33% (4/12). All segments with fibrosis on conventional LGE images were also identified on high-resolution LGE, and an additional 8 segments had fibrosis evident only on high-resolution LGE—which yielded significant increased prevalence of LV segments with fibrosis (16% versus 13%, *P*<0.01). It is also important to recognize that LGE signal intensity varies in relation to magnetic field strength and that prevalence of LGE has been shown to vary in relation to signal intensity thresholds^73,74^—concepts of substantial importance given that differential diagnostic thresholds have been used to define prevalence of LGE in COVID-focused research (Table S1).^48-52,61,72,75-87^ Last, it should be noted that some groups have reported that LGE can occur in high endurance athletes in the absence of COVID-19 infection,^64,88,89^ raising the possibility that observed patterns might be a consequence of increased LV wall stress, altered myocardial perfusion gradients, or hemodynamic sequelae of athletic competition itself. While the underlying mechanism for this association is uncertain, it is known that the finding of LGE itself does not provide temporal information—highlighting the importance of adjunctive CMR approaches to elucidate time course of myocardial injury.

Parametric mapping, like LGE, is subject to heterogeneity in pulse sequence parameters that can provide an important source of variability with respect to diagnostic yield, in addition to the differences of native T1 with magnetic field strength. For T1 mapping, prior COVID-19 studies have used an array of pulse sequences that vary with respect to saturation/inversion pulse design, sampling interval, and fitting algorithm.^48-50,5-80,51,52,61,72,82-84,86,87^ Similarly, among the T2 mapping studies reported, different pulse sequences, fitting algorithms, and signal equations have been used to estimate decay curves^48,49,51,52,61,72,76-80,82-87^—each of which is capable of impacting the derived T2. Additionally, as is the case for LGE, variable spatial resolution provides a potential source of data heterogeneity of particular importance to the post-COVID athlete, given that endurance and strength-trained athletes can manifest differential LV remodeling^90,91^ and that some studies have reported athletes to manifest increased LV trabeculations^92^ (providing a source of partial voxel admixture of LV blood pool and myocardium). In this context, it is worthwhile noting that prior COVID-19 studies of athletic and nonathletic cohorts have used a variety of pulse sequences, different thresholds for delineation of myocardial tissue substrate abnormalities, and performed CMR at variable time points after COVID-19 (see Table S1). Each of these factors, as well as inherent differences in population characteristics and study design, may contribute to heterogeneity in prevalence of reported myocardial tissue substrate abnormalities.

**Figure 3.**
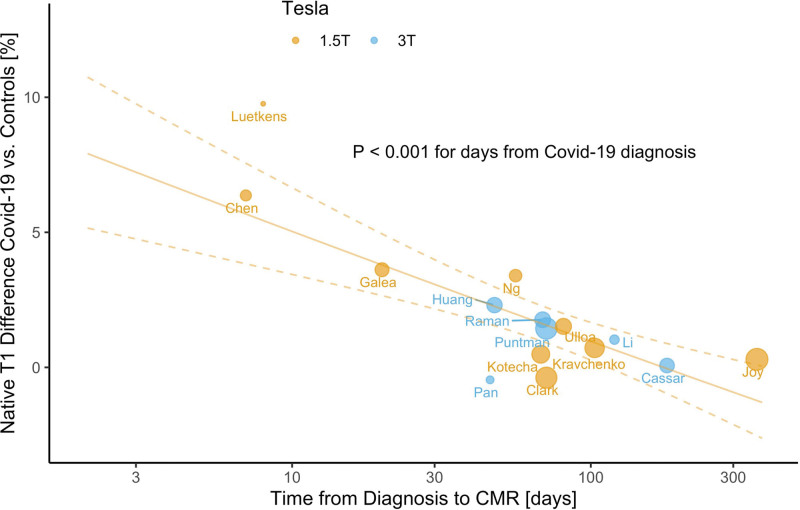
**Meta-regression for percent differences of CMR native T1 in COVID-19 studies.** The percentage difference of the means for native T1 in COVID-19 and control groups regresses with the time since the original COVID-19 diagnosis. The studies selected are for adult, nonathletic cohorts which also include native T1 results for a control group scanned under equivalent conditions (ie, same field strength, scanner, T1 mapping technique). The continuous line and dashed lines correspond to the predicted mean and confidence intervals obtained from a meta-regression model for the ratio of means of native T1 for COVID-19 and control groups. The size of the data points is proportional to the weighs given in the meta-regression analysis. The last name of each study’s first author appears next to the data points. All ratios of means of native T1 were converted to percentage differences for illustration in this figure. The native T1 at the upper bound of its normal range in controls corresponds to ≈5% relative to its mean.^22,48-50,75,76,78,80,83-85,87,93-95^ CMR indicates cardiac magnetic resonance.

It remains a challenge to reconcile the findings of the various reported studies of COVID-19 infected athletes from different centers as the schemes for data acquisition vary by center and published studies have not included controls with the same scanner and protocol to provide center-specific reference ranges. These studies are therefore not consistent with best practice recommendations from CMR expert panels on T1/T2 mapping.^28^ One approach to overcome the limited comparability of T1/T2 parameters could be to consider relative changes of T1/T2 with respect to the center-specific results in healthy controls. This still leaves open the question to what degree controls should match clinical characteristics of COVID-19 patients with respect to key indices (eg, age, gender, cardiovascular disease risk factors)—some studies have expanded additional efforts to address this issue.^50^ Figure 3 illustrates an example of an approach to standardize the results for native T1 differences based on published studies that include T1 reference values from their own center.^22,48-50,75,76,78,80,83-85,87,93-95^ This type of meta-analysis can identify factors, such as the time of recovery because time from infection onset is a source of disease-related variability of native myocardial T1 in COVID-19 survivors.

## When to Consider CMR Testing in the RTP Assessment

Evidence suggests that athletes with cardiac symptoms, severe acute COVID-19 infection requiring hospitalization, or abnormalities on triad testing may benefit from CMR imaging.^63^ Although CMR is an invaluable diagnostic tool in the setting of clinically suspected myocarditis, available evidence does not support its widespread use as a primary screening tool following COVID-19 infection among competitive athletes.^96^ This recommendation is based on an appraisal of the fundamental characteristics of a good screening test. Effective screening tests should be easy to administer, inexpensive, reliable, valid, and should address a disease process that represents a significant public burden. Compared with other forms of screening testing, CMR is generally somewhat more expensive and available largely at tertiary care or academic medical centers.^97^ These limitations render CMR an impractical screening test for the large number (potentially thousands if not more) of competitive athletes who contract COVID-19. Further, as noted above, there is a need for standardized CMR analyses, contextual interpretation of available literature, and need for further study of the prognostic significance of CMR findings including parametric mapping abnormalities in isolation. Available clinical surveillance data from prospective registries as summarized above suggest exceptionally low rates of adverse events among athletes evaluated by triad testing in isolation thereby suggesting that abnormalities detected only by CMR (without symptoms or triad testing abnormalities) are likely of little clinical relevance.

## What Should Constitute a Positive CMR for Myocarditis

As noted in this statement, CMR abnormalities that could be consistent with evidence of persistent myocardial inflammation or myocardial scarring from prior inflammation are derived from (1) parametric mapping results above the upper limit of normal for that specific acquisition sequence and field strength and (2) LGE. LGE can be conceived as (1) likely unrelated and of questionable pathological significance (such as in interventricular insertional LGE), (2) likely unrelated but pathological chronic patterns (prior subendocardial LGE in an ischemic pattern or prior high signal intensity LGE observed in the context of a wall thickening pattern suggestive of hypertrophic cardiomyopathy (HCM)), or (3) likely related acute LGE patterns attributable to recent COVID-19 (subepicardial LGE or mid-myocardial LGE particularly involving the nonseptal walls) colocalized to parametric abnormalities. In this context, CMR findings should be interpreted with this qualification scheme in mind and should fall into one of the following adjudications: (1) no myocarditis (normal native T1, T2, ECV, and no LGE), (2) possible myocarditis (abnormal native T1 or T2, normal ECV, and present but nonspecific LGE), and (3) probable myocarditis (abnormal native T1 and T2 or abnormal ECV, or presence of LGE in a pattern consistent with acute myocarditis and colocalized to parametric abnormalities). It is important to reiterate that available outcomes data for mapping techniques are limited relative to more rigorously validated imaging biomarkers (such as cine structural and functional parameters or LGE) and that clinical decision-making is best predicated upon the latter, more highly substantiated parameters.

## Use of CMR in Follow-Up Imaging

Consideration of the utilization and timing of repeat CMR among athletes with prior abnormal CMR testing is well described in the aforementioned Expert Consensus Decision Pathway document.^63^ In the context of inconsistent or conflicting testing results, a shared decision-making model is reasonable to balance the potential risk of athletic endeavors with the implications of cessation of activity. Moreover, in the competitive athlete, cessation of athletic activity may have significant psychological, financial, and educational implications. The ultimate decision to compete or restrict must be individualized and will depend in part on the quality and significance of the abnormal testing, risk tolerance, and the benefits of competition.

## Research Priorities and Unanswered Questions

This effort to consolidate expert opinions in this statement was motived by the observed variability in utilization and interpretation of CMR in the clinical practice of the RTP assessment. With the exception of the larger registry studies, recommendations put forth here are drawn largely upon presently available case series and cohorts that do not in themselves confer sufficient evidence on which to base definitive recommendations. Furthermore, there is considerable overlap in the CMR imaging applications of the RTP recommendation with assessment of nonathletes with symptoms of PASC.^63^ For this reason, investigations funded by the significant $1.1 billion investment committed by National Institutes of Health to address PASC will likely inform the interpretation of testing results, including CMR, in the RTP assessment. Similarly, CMR features of myocarditis following mRNA COVID-19 vaccine is another area of overlap. A more thorough discussion of CMR in the assessment of PASC or post-mRNA vaccine myocarditis is beyond the scope of this document. Table 3 reflects important knowledge gaps relevant to CMR in the RTP (and PASC) assessment which will be addressed through future investigations leveraging various study designs, registries, and multicenter observational studies.

**Table 3.**
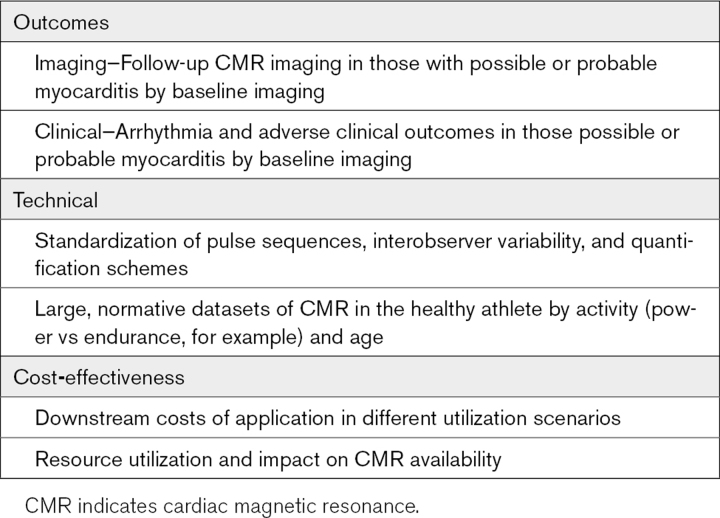
CMR in COVID-Related Myocarditis Research Priorities

## Conclusions

The vast numbers of recreational, collegiate, and professional athletes infected by COVID-19 have placed many clinicians in the unenviable position of rendering clinical clearance for resumption of athletic activities and mitigation of adverse cardiac risk. CMR is an indispensable tool to identify myocardial inflammation owing to COVID-19 infection and current literature suggests that CMR should be applied judiciously in selected cases of symptomatic COVID-19 and abnormal triad testing. Future studies will further inform the prognostic significance of the diversity of reported CMR findings to shape clinical action taken.

## Article Information

### Sources of Funding

None.

### Disclosures

Dr Ruberg reports Research grant support from NIH/National Heart, Lung, and Blood Institute (NIH/NHLBI; R01HL139671), Alnylam Pharmaceuticals, Akcea Therapeutics, and Pfizer, and consulting support from Attralus. Dr Baggish has received funding from the NIH/NHLBI, the National Football Players Association, and the American Heart Association to study issues relevant to this manuscript and receives compensation for his role as team cardiologist from the US Olympic Committee/ US Olympic Training Centers, US Soccer, US Rowing, the New England Patriots, the Boston Bruins, the New England Revolution, and Harvard University. Dr Hays reports Research grant support from NIH/NHLBI (1R01HL147660). Dr Jerosch-Herold reports Research grant support from NIH/National Institute on Aging (NIA; 1R01AG052282). Dr Kim reports Research grant support from NIH/NHLBI (R01HL159055). Dr Ordovas reports Research contract from NIH/National Institute of Biomedical Imaging and Bioengineering (NIBIB; 75N9202D00018) and Research grant from American College of Radiology Fund for Collaborative Research in Imaging (FCRI) titled: Transcatheter Aortic Valve Replacement (TAVR) Registry. C. Shenoy reports Research grant support from NIH/NHLBI (K23HL132011) and University of Minnesota Clinical and Translational Science Institute K-R01 Transition to Independence Grant (supported by the NIH grant UL1TR002494). Dr Weinsaft reports Research support from NIH/NHLBI (R01HL151686, R01HL159055), General Electric Healthcare. Dr Woodard reports Research support from NIH/NHLBI (R01HL150891, R01HL142297), NIH/National Center for Advancing Translational Sciences (NCATS; UL1TR002345), NIH/NIBIB (T32EB021955), Siemens, Bayer. The other author reports no conflicts.

### Supplemental Material

Table S1

## Supplementary Material
